# Translational approach to address therapy in myotonia permanens due to a new *SCN4A* mutation

**DOI:** 10.1212/WNL.0000000000002721

**Published:** 2016-05-31

**Authors:** Jean-François Desaphy, Roberta Carbonara, Adele D'Amico, Anna Modoni, Julien Roussel, Paola Imbrici, Serena Pagliarani, Sabrina Lucchiari, Mauro Lo Monaco, Diana Conte Camerino

**Affiliations:** From the Departments of Biomedical Sciences and Human Oncology (J.-F.D.) and Pharmacy & Drug Sciences (R.C., J.R., P.I., D.C.C.), University of Bari Aldo Moro, Bari; Unit of Neuromuscular and Neurodegenerative Disorders (A.D.), Bambino Gesù Children's Hospital, Rome; Departments of Geriatrics, Neurosciences, and Orthopedics (A.M., M.L.M.), Institute of Neurology, Catholic University of the Sacred Heart, Rome; Dino Ferrari Centre (S.P., S.L.), Neuroscience Section, Department of Pathophysiology and Transplantation (DEPT), University of Milan; and Neurology Unit (S.P., S.L.), IRCCS Foundation Ca' Granda, Ospedale Maggiore Policlinico, Milan, Italy.

## Abstract

**Objective:**

We performed a clinical, functional, and pharmacologic characterization of the novel p.P1158L Nav1.4 mutation identified in a young girl presenting a severe myotonic phenotype.

**Methods:**

Wild-type hNav1.4 channel and P1158L mutant were expressed in tsA201 cells for functional and pharmacologic studies using patch-clamp.

**Results:**

The patient shows pronounced myotonia, slowness of movements, and generalized muscle hypertrophy. Because of general discomfort with mexiletine, she was given flecainide with satisfactory response. In vitro, mutant channels show a slower current decay and a rightward shift of the voltage dependence of fast inactivation. The voltage dependence of activation and slow inactivation were not altered. Mutant channels were less sensitive to mexiletine, whereas sensitivity to flecainide was not altered. The reduced inhibition of mutant channels by mexiletine was also observed using clinically relevant drug concentrations in a myotonic-like condition.

**Conclusions:**

Clinical phenotype and functional alterations of P1158L support the diagnosis of myotonia permanens. Impairment of fast inactivation is consistent with the possible role of the channel domain III S4-S5 loop in the inactivation gate docking site. The reduced sensitivity of P1158L to mexiletine may have contributed to the unsatisfactory response of the patient. The success of flecainide therapy underscores the usefulness of in vitro functional studies to help in the choice of the best drug for each individual.

Gain-of-function missense mutations of the skeletal muscle Nav1.4 sodium channel cause myotonia or flaccid weakness. Impaired fast-inactivation of hNav1.4 mutants likely induces myotonia, while enhancement of activation and impaired slow inactivation contribute to paralytic attacks.^1^ Various substitutions at the same amino acid (Gly1306) induce various degrees of channel alteration, which correlate to the severity of symptoms ranging from the mild myotonia fluctuans (G1306A) to the severe myotonia permanens (G1306E).^2^ Unusual phenotype is also explained by specific behavior of channel mutant. Carriers of the P1158S mutation show myotonia in a warm environment and paralytic attacks at cold temperatures.^3^ Accordingly, heterologously expressed P1158S channels show alteration of activation and slow inactivation at 22°C but not 37°C.^4,5^

The sodium channel blocker mexiletine is the preferred drug to alleviate myotonia.^6^ Nevertheless, in case of side effects or lack of efficacy, mexiletine can be substituted for by another sodium channel blocker. We previously demonstrated that flecainide is successful in mexiletine-low responsive patients carrying G1306E, likely due to mutation-specific gating changes.^7–9^

We report the clinical, functional, and pharmacologic characterization of a new hNav1.4 mutation, p.P1158L, found in a severely affected girl with symptoms resembling myotonia permanens. Gating alteration of heterologously expressed P1158L channels is consistent with the phenotype. The patient remained unsatisfied with mexiletine, but claimed great improvement with flecainide. Accordingly, P1158L channels are less sensitive to mexiletine in vitro but show unaltered flecainide sensitivity. These results highlight how in vitro pharmacologic study of hNav1.4 mutants can help in better addressing treatment in myotonic patients.

## METHODS

### Genetic analysis.

After written informed consent, genomic DNA was extracted from peripheral blood. DM1 genetic analysis was performed following the standard diagnostic method. All the coding exons and intron–exon junctions of *SCN4A* and *CLCN1* were amplified (primer sequences and conditions are available upon request). The PCR fragments were directly sequenced using Big Dye Terminator Cycle Sequencing Kit in an automated sequencer 3130 (Applied Biosystems, Foster City, CA). SeqScape software (Applied Biosystems) was used to align and compare the sequences with National Center for Biotechnology Information control sequences (NG_011699 and NM_000334 for *SCN4A*; NG_009815 and NM_000083 for *CLCN1*). To confirm the result obtained on exon 19 of *SCN4A*, amplification and sequencing were repeated and the variant was checked in the father.

### Mutagenesis and expression of recombinant sodium channels.

The pRc/CMV-hNav1.4 vector encoding the wild-type (WT) skeletal muscle voltage-gated sodium channel was provided by Desaphy et al.^7^ The P1158L mutant was engineered using the QuikChange Lightning Site-Directed Mutagenesis kit (Agilent Technologies, Santa Clara, CA) and was confirmed by complete sequencing. The WT or P1158L hNav1.4 plasmids (1 µg/100-mm dish) were transiently coexpressed in tsA201 cells with the pCD8-IRES-hβ1 plasmid containing the CD8 receptor and the auxiliary sodium channel β1 subunit (0.5 µg/100-mm dish), using the calcium phosphate method.^7^ Microbeads coated with anti-CD8 antibody (Dynal-Invitrogen, Milan, Italy) were used to identify cells for patch clamp experiments.

### Whole-cell recording and data analysis.

Sodium currents (*I*_Na_) were recorded in whole-cell patch-clamp configuration at room temperature (20°C–22°C), as previously described.^10^ The pipette solution contained (in mM) 120 CsF, 10 CsCl, 10 NaCl, 5 EGTA, and 5 Cs-HEPES (pH 7.2). The bath solution contained (in mM) 150 NaCl, 4 KCl, 2 CaCl_2_, 1 MgCl_2_, 5 Na-HEPES, and 5 glucose (pH 7.4). Patch pipettes had resistance ranging from 1 to 3 MΩ. Capacitance currents were partially compensated using the amplifier circuit. Only those data obtained from cells exhibiting series resistance errors <5 mV were considered for analysis.^10^

Mexiletine-HCl and flecainide-acetate salt (Sigma-Aldrich, Milan, Italy) were solubilized in bath solution at the final concentration. The patched cell was exposed to a continuous stream of control or drug-supplemented bath solution. A maximum of 2 drug concentrations were tested on each cell, to minimize the possible bias due to *I*_Na_ rundown, as previously described.^10,11^ Because of the known spontaneous shift of voltage dependence during whole-cell experiments, much care was taken to perform the various protocols respecting a constant sequence to allow comparison between the cells.

Data analysis was performed using pCLAMP 10.3 (Axon Instruments, Union City, CA) and SigmaPlot 8.02 (Systat Software GmbH, Erkrath, Germany).^10,11^ The *I*_Na_ decay was fit to a single exponential function, 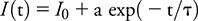 (equation 1), to determine the inactivation time constant τ, which is reported as mean ± SEM from n cells. To plot the voltage dependence of activation, the sodium conductance *G*_Na_ was calculated from the peak *I*_Na_ amplitude using the theoretical reversal potential for sodium ions (*E*_Na_ = +68.4 mV). The voltage-dependent relationships of activation and fast inactivation were fit to a Boltzmann function, 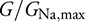 or 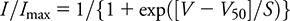 (equation 2), where *V*_50_ is the midpoint and *S* is the slope factor. Because slow inactivation is not complete, a nonzero residual current (*I*_R_) was introduced in the function 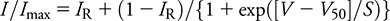 (equation 3). The complete voltage dependence relationships were obtained in each cell, and the fit parameters were averaged as mean ± SEM from n cells to allow statistical analysis using unpaired Student *t* test, with *p* < 0.05 considered as significant. The concentration-response curves were obtained by combining data obtained in different cells at various drug concentrations, reporting each data point as the mean ± SEM from n cells. The relationships were fit to a first-order binding function 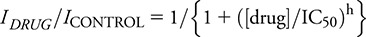 (equation 4), where IC_50_ is the half-maximum inhibitory concentration and h is the logistic slope factor. The fit parameter values are reported together with the SE of the regression.

## RESULTS

### Case report.

The proband was born in Algeria of consanguineous parents (the paternal grandmother and the maternal great-grandmother were first-line cousins) coming from a village of nearly 3,000 inhabitants (figure e-1 on the *Neurology*® Web site at Neurology.org). She did not require resuscitation at birth, but her mother noticed feeding difficulties in the neonatal period, facial grimaces during crying, and convergent strabismus. When she was 9 years of age, an Algerian pediatrician reported generalized muscle hypertrophy with facial dysmorphism, including narrow palpebral fissures and small mouth. Short and hypertrophic neck, slender hands, flat feet, and kyphoscoliosis were described together with statural and ponderal growth delay. CK level was elevated (about 600 UI/L). EMG showed subcontinuous myotonic discharges. Echocardiogram was unremarkable. The patient was diagnosed with Schwartz-Jampel disease. She was given carbamazepine for 2 years without any referred clinical benefit.

The girl came to our observation when she was 11 years old. Muscle hypertrophy was widespread but more evident in facial muscles, neck, and upper limbs muscles. Shoulder girdle and biceps brachii hypertrophy was responsible for humeral intrarotation attitude and elbow retraction. Finger flexors myotonic stiffness hindered hands opening. Voluntary and percussion myotonia at hands lasted more than 5 seconds. Eyelid myotonia was striking with marked lid lag and convergent strabismus. Extrinsic eye muscles myotonia did not allow her to follow the examiner's finger by eyes. Tongue myotonia was so pronounced that she could not oscillate the protruded tongue and swallowing was slow. No muscle weakness was observed or reported, except for orbicularis oculi. Muscle stiffness was severe and exacerbated by cold temperatures, limiting all daily activities. EMG showed continuous repetitive myotonic discharges in absence of myopathic changes. No significant compound muscle action potential amplitude changes were observed upon 3-Hz repetitive nerve stimulation.^12^

Family history was remarkable for the proband father, who showed muscle stiffness, facial muscle weakness, including orbicularis oculi and oris, mild symmetrical palpebral ptosis, distal and semidistal limb muscles weakness (3.5 score on the Medical Research Council scale), and muscle hypotrophy. There was neither facial muscle myotonia nor lid lag phenomenon. Voluntary and percussion myotonia at hands lasted for more than 5 seconds. EMG revealed myopathic changes and myotonic runs. These data were compatible with the diagnosis of myotonic dystrophy type 1 (DM1), which was confirmed by genetic analysis with the presence of 250 CTG repeats at the DMPK locus.

The proband was analyzed for both DM1 and *SCN4A* mutations. The genetic analysis showed the presence of 310–390 CTG repeats and a new *SCN4A* mutation in exon 19 (c.3473C>T, p.Pro1158Leu), indicating the coexistence of DM1 and sodium channel myotonia. The *SCN4A* mutation was absent in the father, while the mother was not available for genetic analysis. Nevertheless, no clinical sign of myotonia was found in the mother, thereby suggesting that p.P1158L was a de novo mutation in the proband. The p.Pro1158Leu was not described in public databases (i.e., ExAC and 1000 G) and was absent in 140 alleles from North African controls and in 350 alleles from Italian controls. Bioinformatic analysis using Polyphen2 and Mutation Taster prediction software classified the variant as probably damaging and disease-causing, respectively. Sequence analysis excluded pathogenic mutations in the *CLCN1* gene.

After medical evaluation, the proband started 100 mg mexiletine 2 times a day. After 15 days, she discontinued mexiletine because of side effects (gastrointestinal pain and referred loss of hair) and, according to her parents, poor improvement of stiffness. Flecainide was started 35 mg 2 times a day. Upon clinical examination, both drug treatments reduced tongue and eye myotonia. Watching the video done before and during either treatment, it was difficult to appreciate quantitative differences in the antimyotonic effects of the 2 drugs. However, the proband claimed a striking improvement of myotonia with flecainide compared to mexiletine. Also, the father declared a major myotonia improvement and minor side effects with flecainide.

### Functional characterization of P1158L hNav1.4 mutant.

**Figure 1:**
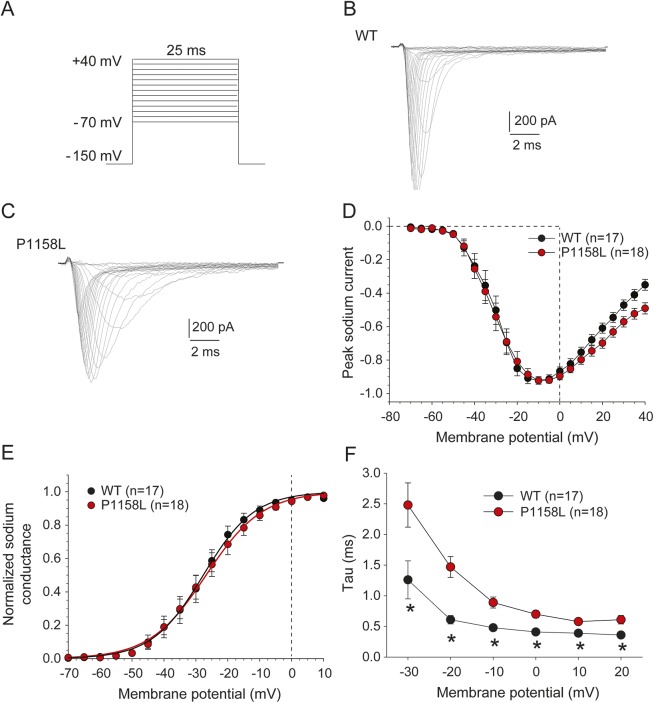
Families of sodium currents generated by wild-type (WT) and P1158L hNav1.4 channels (A) The voltage clamp protocol consisted of 25-ms test pulses ranging from −70 to +40 mV, applied in 10-mV increments from the holding potential of −150 mV. (B) Representative family of WT hNav1.4 currents. (C) Representative family of P1158L hNav1.4 currents. (D) The current-voltage relationships of P1158L and WT were well superimposed. (E) The sodium conductance (*G*_Na_) was calculated from measured peak *I*_Na_ currents and theoretical reversal potential for sodium ions (*E*_Na_ = +68.4 mV). Resulting *G*_Na_ values were normalized to the maximal conductance and plotted as a function of voltage. The relationships, fitted using a Boltzmann function (equation 2 in Methods), were superimposed; fit parameters values are given in the table. (F) The *I*_Na_ decay was fitted to an exponential function (equation 1 in Methods) and the time constant, τ, is reported as a function of voltage. Between −30 and +20 mV, τ was significantly longer for P1158L compared to WT (at least *p* < 0.05 with unpaired Student *t* test).

Whole-cell sodium currents generated by transiently expressed P1158L and WT channels were recorded in tsA201 cells using patch-clamp (figure 1, A–C). The current-voltage relationships of P1158L and WT were well superimposed (figure 1D), and the voltage dependence of activation was similar (figure 1E, table). Nevertheless, the P1158L currents show a slowing of inactivation, with a significant increase of the decay time constant for P1158L currents (figure 1F). Such a defect is commonly observed in myotonic Nav1.4 mutations and likely contributes to the disease manifestation.^7,13^

**Table:**
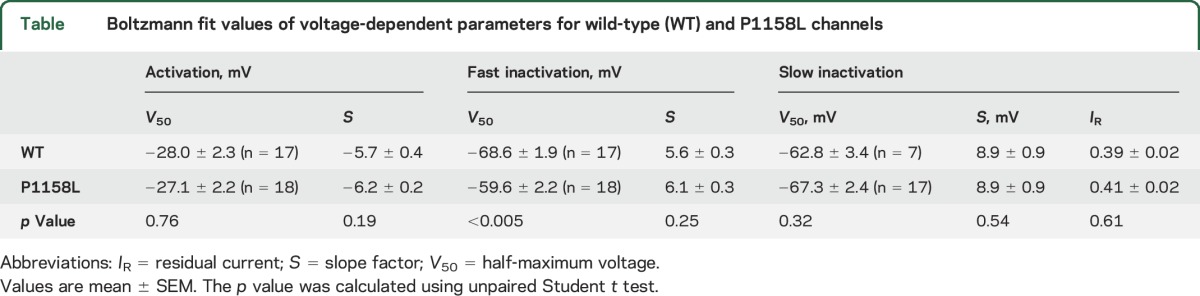
Boltzmann fit values of voltage-dependent parameters for wild-type (WT) and P1158L channels

**Figure 2:**
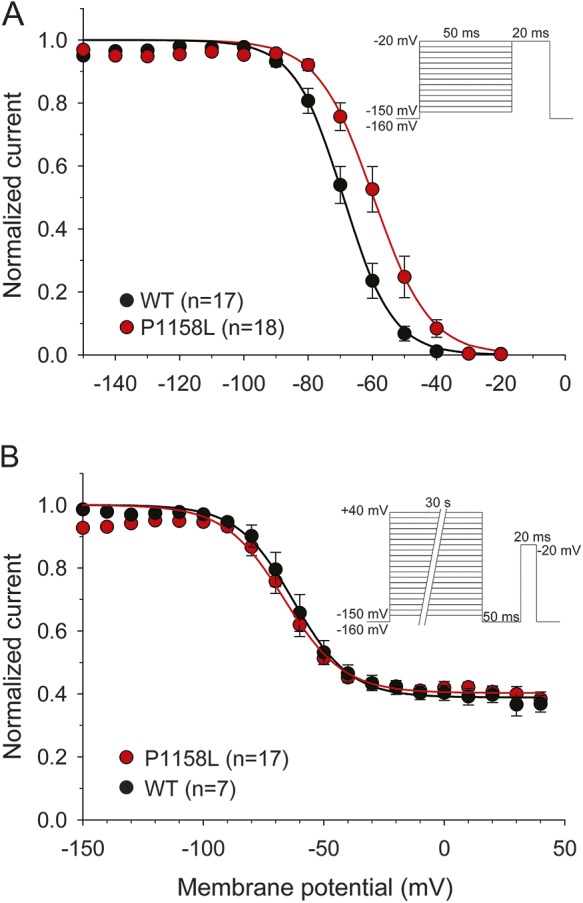
Voltage dependence of fast and slow inactivation of wild-type (WT) and P1158L hNav1.4 channels (A) To plot fast inactivation voltage dependence, sodium currents measured during a test pulse at −20 mV were reported as a function of the conditioning pulse ranging from −150 to −20 mV, applied in 10-mV increments (inset). The relationships, fitted to a Boltzmann function (equation 2 in Methods), was positively shifted by 9 mV for P1158L compared to WT. (B) Slow inactivation was induced by 30-seconds-long conditioning pulses. Fast inactivation was removed by an intermediate 50-ms-long pulse at −160 mV, before assessing channel availability at −20 mV (inset). A fraction of channels do not enter slow inactivation. The relationships of WT and P1158L channels, fitted using a Boltzmann equation (equation 3 in Methods), were well superimposed. Fit parameter values are given in the table.

The voltage dependences of fast and slow inactivation were studied using conventional 2-pulse voltage protocols (figure 2). The normalized peak sodium current amplitude measured during the test pulse was reported as a function of conditioning pulse voltage. The relationships were fit to a Boltzmann equation (table). The P1158L mutation induced a significant positive shift of the half-maximum inactivation potential by 9 mV. Such a shift is a likely mechanism contributing to aberrant sarcolemma excitability. Conversely, the voltage dependence of slow inactivation of P1158L was superimposed to that of WT.

### Pharmacologic characterization of P1158L Nav1.4 mutant.

**Figure 3:**
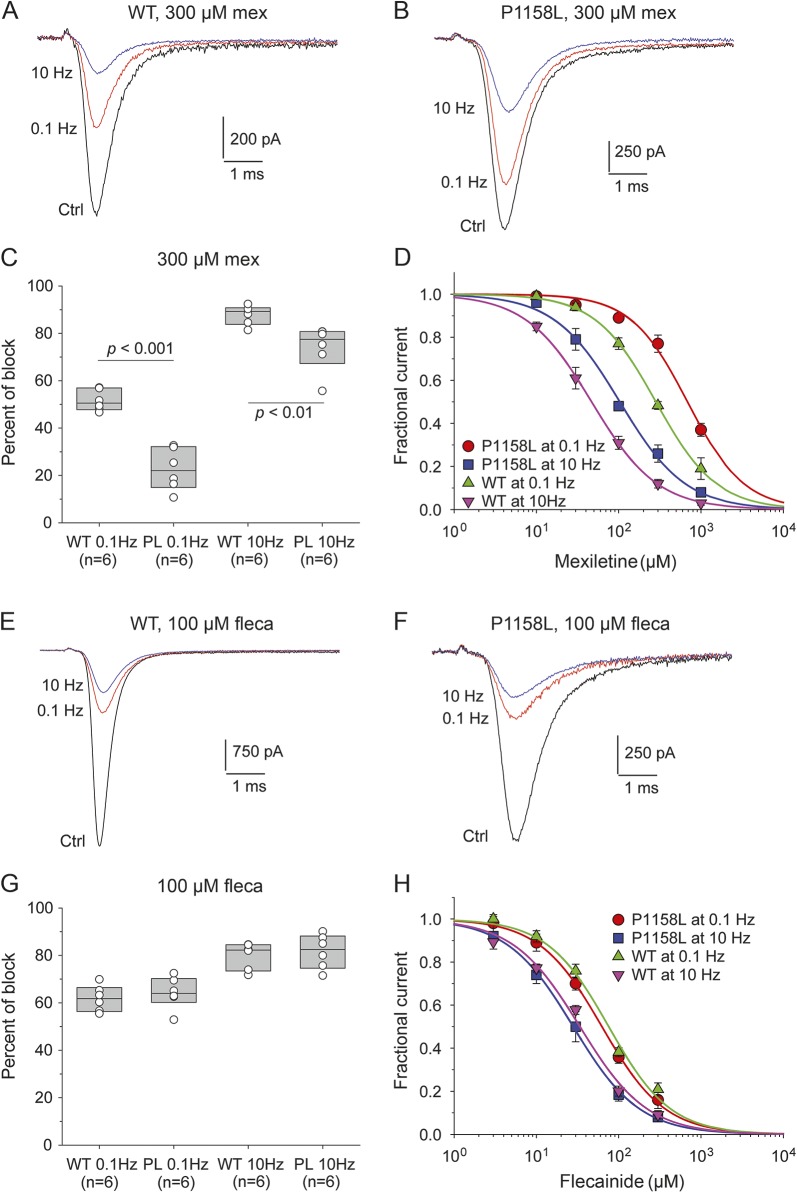
Effects of mexiletine and flecainide on wild-type (WT) and P1158L hNav1.4 channels (A, B) Representative WT and P1158L current traces recorded at steady-state before and during application of 300 µM mexiletine at 0.1 and 10 Hz stimulation frequencies. (C) Box and whisker chart representation of mean effects of 300 µM mexiletine at both stimulation frequencies. Using unpaired Student *t* test, inhibition of P1158L channels was significantly lower compared to WT. (D) The concentration-effect relationships for mexiletine on WT and P1158L, fitted to a first-order binding function (equation 4 in Methods), confirmed the lower sensitivity of P1158L channels to mexiletine. (E, F) Representative WT and P1158L current traces recorded at steady-state before and during application of 100 µM flecainide at 0.1 and 10 Hz stimulation frequencies. (G) Box and whisker chart representation of mean effects of 100 µM flecainide at both stimulation frequencies. No significant difference was found between WT and P1158L channels. (H) The concentration-effect relationships for flecainide on WT and P1158L, fitted to a first-order binding function, were superimposed.

To allow comparison of channel pharmacology to previous data,^8^ we measured inhibition of *I*_Na_ elicited at −30 mV from an holding potential of −120 mV, every 10 (0.1 Hz) or 0.1 (10 Hz) seconds. Mexiletine (300 µM) produced a minor inhibition of P1158L *I*_Na_ at 0.1 Hz (−56.1%, n = 6, *p* < 0.001) and 10 Hz (−16.0%, n = 6, *p* < 0.01) compared to WT (figure 3, A–C). The concentration-response relationships were shifted toward greater mexiletine concentrations for P1158L channels (figure 3D). At 0.1 Hz, the half-maximum inhibitory concentration (IC_50_ ± SE of the regression) of mexiletine was 283 ± 5 µM for WT and 686 ± 56 µM for P1158L; At 10 Hz, the IC_50_ was 47 ± 1 µM for WT and 102 ± 7 µM for P1158L.

Flecainide was tested using the same protocol. Similarly to previous results,^8^ 100 µM flecainide produced ∼60% inhibition of *I*_Na_ at 0.1 Hz and ∼80% inhibition at 10 Hz (figure 3, E–G). Flecainide effects on P1158L channels were not statistically different from those exerted on WT. At 0.1 Hz, the IC_50_ was 76 ± 7 µM for WT and 63 ± 2 µM for P1158L; at 10 Hz, it was 34 ± 4 µM for WT and 28 ± 1 µM for P1158L (figure 3H).

**Figure 4:**
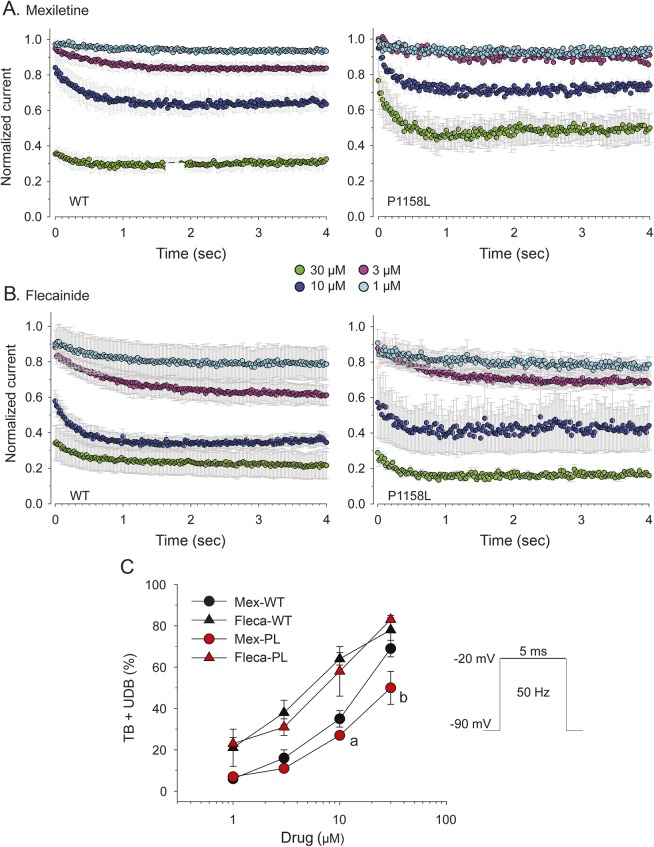
Effects of mexiletine and flecainide on sodium currents in a myotonia-like condition Mexiletine and flecainide were tested on hNav1.4 channels in a condition more similar to a myotonic discharge of action potentials. The holding potential was set to −90 mV; the test pulse lasted 5 ms and was applied at 50 Hz frequency. (A, B) Time course of use-dependent *I*_Na_ inhibition (UDB) induced at 50 Hz by various concentrations of mexiletine or flecainide. The use-dependent *I*_Na_ reduction recorded in absence of drug (∼38%) was subtracted to show the net effect of drugs. The data point at time zero corresponds to tonic block (TB) measured at 0.1 Hz stimulation. Each time course is the mean ± SEM from 3 to 7 cells. (C) Concentration-effect relationships for steady-state TB + UDB exerted by mexiletine and flecainide on wild-type (WT) and P1158L currents. Flecainide was more potent than mexiletine on either channel variant. Mexiletine, 10–30 µM, was less potent on P1158L compared to WT channels (at least *p* < 0.05 with unpaired Student *t* test). Flecainide effects were similar for WT and P1158L channels.

We also tested the drugs in a condition more similar to a myotonic discharge of action potentials: the holding potential was −90 mV, the test pulse lasted 5 ms and was applied at 50 Hz frequency.^14^ In absence of drug, such a protocol induced a use-dependent reduction (UDR) of *I*_Na_ amplitude by 38 ± 3% (n = 34, not shown). The drugs were applied to *I*_Na_ first during 0.1 Hz stimulation to develop tonic block (TB), then during 200 test pulses at 50 Hz to measure the use-dependent block (UDB). The net effect of drug was calculated offline by subtracting UDR from UDB and further adding TB, as previously detailed.^14^ The time course of net drug effect on WT and P1158L *I*_Na_ is shown in figure 4, A and B. The data point at time zero represents TB, which increased with drug concentration. During the following sweeps at 50 Hz, the *I*_Na_ amplitude decreased to a steady state. Inhibitory effect of 10 and 30 µM mexiletine was significantly less pronounced on P1158L channels compared to WT, resulting in a left shift of the concentration-response curve for P1158L (figure 4, A and C). In contrast, little difference was found between the 2 channels for flecainide (figure 4, B and C).

## DISCUSSION

Clinically, the proband's phenotype was comparable to myotonia permanens, which is characterized by severe myotonia aggravated by cold, onset near birth with possible breathing difficulties, and subcontinuous myotonic EMG.^15^ Two other *SCN4A* mutations have been linked to myotonia permanens: G1306E and Q1633E.^15,16^ Patients with more severe cases may encounter life-threatening severe neonatal episodic laryngospam.^16–19^ Conversely to her father, the proband did not show clinical manifestation related to myotonic dystrophy except for the orbicularis oculi weakness. Neither weakness nor EMG myopathic changes were detected in other muscles. Both mexiletine and flecainide were ineffective in changing this clinical sign. Yet we cannot exclude that the DM1 and *SCN4A* mutations may compound to increase the severity of her myotonia, as recently suggested.^20,21^

The P1158L mutation found in the proband is located in the intracellular linker between the fourth and fifth segment of domain III of hNav1.4. Another mutation at this position, P1158S, is associated with myotonia in warm environment and paralytic attacks in cold conditions.^3^ The P1158S channel shows normal behavior at 37°C and alteration of activation and slow inactivation at 22°C.^4,5^ Other mutations close to Pro1158 include A1152D associated with paramyotonia congenita^22^ and I1160V linked to sodium channel myotonia^23,24^ or paramyotonia congenita (Lo Monaco, unpublished data). Those mutations affect the fast inactivation and deactivation of the channel. Indeed, the short S4-S5 loop of domain III is thought to form the docking site of the DIII-DIV loop acting as the inactivation gate.^25^ Accordingly, we found that P1158L induced a slower rate of current decay and a depolarized shift in the voltage dependence of availability, which are common defects associated with sodium channel myotonia, while the lack of effect on activation and slow inactivation is consistent with the lack of paralytic episodes in the patient.^1^ Although proline and leucine are both hydrophobic residues, proline cannot form hydrogen bonding with its embedded backbone nitrogen. Thus the substitution by leucine likely modifies the architecture of the S4-S5 loop. The introduction of the hydrophilic serine may determine a different alteration of the loop, which may explain the different effects of P1158L and P1158S on channel behavior and consequently on clinical phenotype. Importantly, P1158L produced channel defects similar to those of the 2 other myotonia permanens mutations.^7,16^ The rightward shift of the midpoint of fast inactivation voltage dependence was 9, 10.7, and 12.6 mV for P1158L, Q1633E, and G1306E, respectively. In addition, the current decay was greatly slowed by all 3 mutations.

Although the proband showed a partial response to mexiletine upon clinical examination, she complained of general discomfort and asked for treatment interruption. We and others have previously demonstrated that myotonic mutations can affect the effect of mexiletine, either due to altered intrinsic affinity or to mutation-induced altered gating.^7,26^ By inducing a leftward shift of channel availability voltage dependence, the myotonia permanens G1306E mutation is less sensitive to mexiletine compared to WT channels.^7^ We further demonstrated that, instead, the mutation did not affect the sensitivity to flecainide, and this eventually allowed a successful shift of therapy from mexiletine to flecainide in a mother and her son, both carrying G1306E and showing a very limited response to mexiletine.^8,9^ We also recently demonstrated in a pharmacologically induced rat model of myotonia that flecainide is an efficient antimyotonic drug at clinically relevant doses.^14^ On the basis of this experience, the P1158L carrier was given flecainide. She remained very satisfied, claiming an attenuation of stiffness incomparably greater and more stable than with mexiletine.

In vitro, P1158L channels showed a reduced affinity to mexiletine compared to WT. As for G1306E,^8^ we can hypothesize that the reduced affinity is, at least in part, due to the rightward shift of the voltage dependence of fast inactivation: the minor proportion of inactivated P1158L channels at the holding potential, compared to WT, may reduce the apparent affinity of mutant channels to mexiletine. Blockade of P1158L channels by mexiletine is also reduced in a myotonic-like condition at the clinically relevant drug concentration of 10 µM. The human therapeutic blood concentration range is 4–11 µM for mexiletine and 0.5–2 µM for flecainide.^27^ In contrast, the mutation did not significantly alter the block exerted by flecainide.

In this study, we demonstrate that the new P1158L hNav1.4 mutation is likely responsible for myotonia. Both the clinical phenotype and the biophysical channel defects are consistent with myotonia permanens. The reduced apparent affinity of the mutant channel to mexiletine may have contributed to the unsatisfactory response of the patient to the drug. Together with the previous experience with G1306E carriers,^8,9^ this result suggests that most of the mutations showing a rightward shift of fast inactivation voltage dependence may greatly benefit from flecainide treatment. The success of therapy with flecainide further supports the development of precision medicine for the treatment of myotonic syndromes, in which the functional study of the mutant protein at bench may be helpful to choose the best drug for each individual.^28,29^

## Supplementary Material

Data Supplement

## GLOSSARY

DM1: myotonic dystrophy type 1
IC_50_: half-maximum inhibitory concentration
TB: tonic block
UDB: use-dependent block
UDR: use-dependent reduction
WT: wild-type
