# Supplementary material for: Translational approach to address therapy in myotonia permanens due to a new SCN4A mutation
Source: Neurology. 2016 May 31;86(22):2100–8. doi: 10.1212/WNL.0000000000002721 (PMC4891212; doi:10.1212/WNL.0000000000002721)
Supplement: Data Supplement [file supp_86_22_2100__index.html]

Data Supplement 

# Translational approach to address therapy in myotonia permanens due to a new *SCN4A* mutation

## Data Supplement

One figure; PDF file.

**Neurology® data supplements are not copyedited before publication. Published editorials and translations have been copyedited.  
 © 2016 American Academy of Neurology.  
  
 Files in this Data Supplement:**

- Figure e-1 - PDF file
