# Supplementary material for: Translational approach to address therapy in myotonia permanens due to a new SCN4A mutation
Source: Neurology. 2016 May 31;86(22):2100–8. doi: 10.1212/WNL.0000000000002721 (PMC4891212; doi:10.1212/WNL.0000000000002721)
Supplement: Data Supplement [file supp_WNL.0000000000002721_Figure_e-1.pdf]

Translational approach to address therapy in myotonia permanens due to a new SCN4A mutation

Jean-François Desaphy (PhD), Roberta Carbonara (PhD), Adele D'Amico (MD), Anna Modoni (MD), Julien Roussel (PhD), Paola Imbrici (PhD), Serena Pagliarani (PhD), Sabrina Lucchiari (PhD), Mauro Lo Monaco (MD), Diana Conte Camerino (PhD)

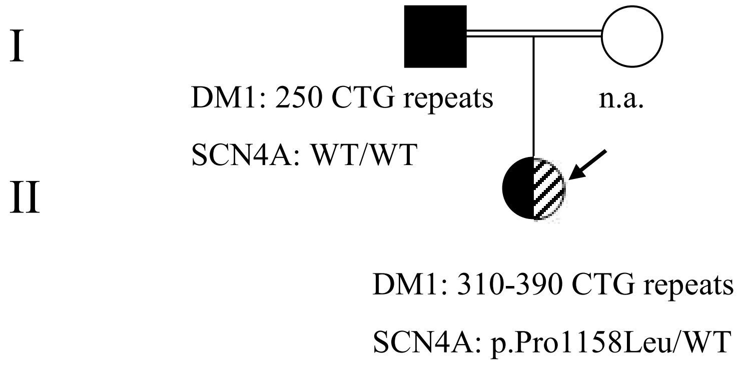

Figure e-1. Family tree. The proband (II-1) is indicated by arrow. Plain symbols indicate the presence of DM1 mutation. The hashed symbol indicates the presence of SCN4A mutation. The mother (I-2) was not available for genetic analysis.
